# Coronary MRI with induced vasodilation using isosorbide dinitrate

**DOI:** 10.1186/1532-429X-11-S1-P5

**Published:** 2009-01-28

**Authors:** Peng Hu, Christian Stoeck, Dana C Peters, Kraig V Kissinger, Beth Goddu, Lois Goepfert, Warren J Manning, Reza Nezafat

**Affiliations:** Beth Israel Deaconess Medical Center, Boston, MA USA

**Keywords:** Isordil, Coronary Image, Isosorbide Dinitrate, Sublingual Nitroglycerin, Healthy Adult Subject

## Introduction

Despite technical progress, coronary magnetic resonance imaging (MRI) still faces multiple challenges. Coronary vasodilators, such as sublingual nitroglycerin (NTG) or longer acting nitrates (e.g., isosorbide dinitrate), are commonly used to study coronary circulation. Terashima et al. [1] reported use of coronary MRI to evaluate the effect of sublingual NTG. Isosorbide dinitrate (Isordil) has been previously reported in a multi-center clinical coronary MRI trial [2], however no data has been provided to quantify coronary MRI image quality improvement and time course of Isordil.

## Purpose

To investigate the impact of Isordil administration on SNR, vessel diameter and overall image quality in coronary MRI.

## Materials and methods

Coronary images were acquired on a cohort of healthy adult subjects before and after Isordil administration. Subjects were divided into four groups to investigate the impact of the imaging sequence and dose. In groups A and B the images were acquired using SSFP imaging sequence with either 2.5 mg or 5 mg Isordil dose. In groups C and D images are acquired using GRE with either 2.5 mg or 5 mg Isordil dose. The impact of vasodilator during a time course was studied by repeated imaging. A free breathing, 3D VCG gated GRE sequence with typical imaging parameters of TR = 7.7 ms, TE = 2.2 ms, FOV = 270 × 270 × 30 mm^3^, flip angle = 30°, spatial resolution of 0.7 × 1 × 1.5 mm^3^ reconstructed to 0.52 × 0.52 × 0.75 mm^3^ was used. The imaging parameters for SSFP imaging included: TR = 4.6 ms, TE = 2.3 ms, FOV = 270 × 270 × 30 mm^3^, flip angle = 90°, spatial resolution of 1 × 1 × 1.5 mm^3^ reconstructed to 0.52 × 0.52 × 0.75 mm^3^ and a half α preparation pulse. T_2_ prep, fat saturation and navigator sequences were used in both sequences. In order to obtain a more accurate SNR measurement, no parallel imaging was used. SNR was measured with a previously published method [3]. To be consistent, the same noise and signal ROI's were used in all the GRE and balanced-SSFP scans during a study, unless motion is detected between the scans. The coronary cross-sectional diameter in the proximal right coronary artery was measured using the Soap Bubble tool (Philips Healthcare, Best, NL).

## Results

**Figure 1:**
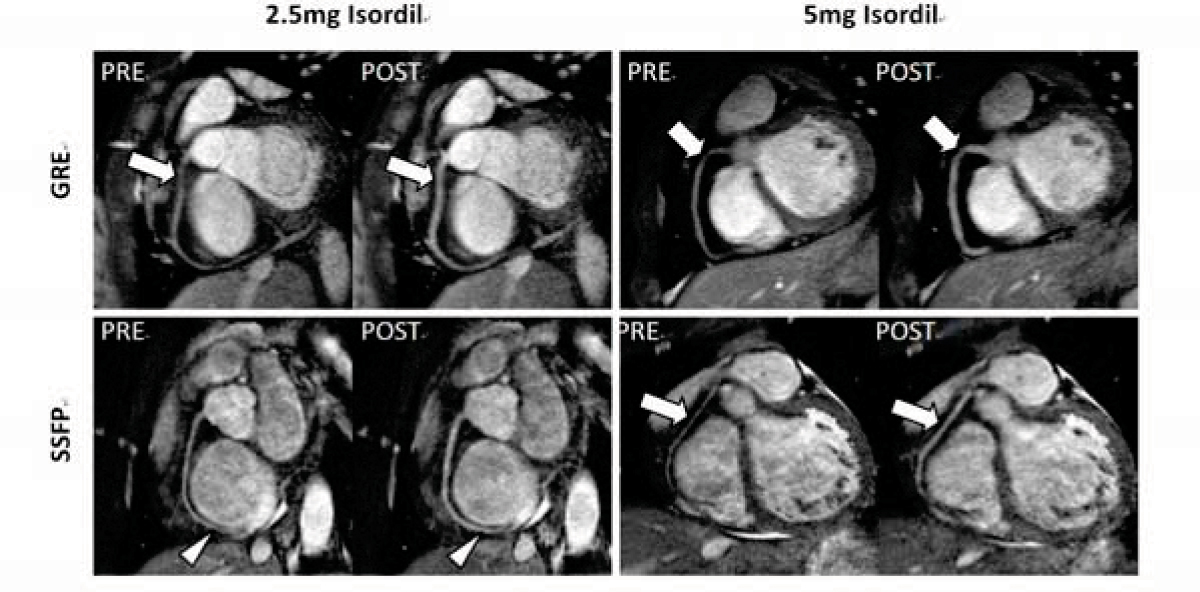
**Examples of pre- and post-Isordil images from four different subjects using combinations of two different sequences (GRE and SSFP) and two different Isordil doses (2.5 mg and 5 mg) at 10–15 minutes after Isordil**.

**Figure 2:**
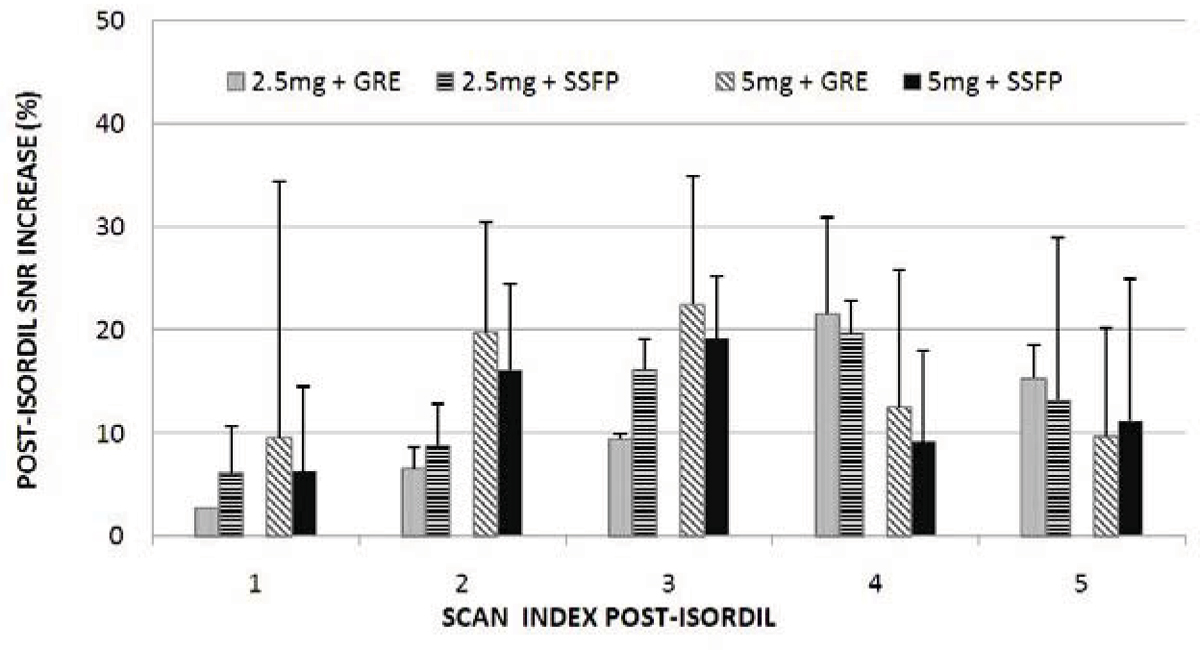
**The time course of SNR increase after Isordil administration using the GRE and SSFP sequences**. The first 5 post-Isordil scans were used to calculate the time course. The post-Isorfil times for the five scans were 1.3 ± 0.7, 10.4 ± 4.2, 17.7 ± 6.7, 26.2 ± 7.3 and 32.3 ± 8.3 minutes.

Figure 1 demonstrates the improved coronary image quality 10–15 minutes after Isordil administration. The vasodilation and signal enhancement help better delineate and differentiate the right coronary artery (arrows). The visibility of distal branches of the coronaries was improved (arrow heads). Figure 2 shows the SNR enhancement during the time course of 5 post-Isordil scans. The maximum SNR increase was 21.5% ± 9.3% for GRE with 2.5 mg dose, 22.5% ± 12.3% for GRE with 5 mg, 19.7% ± 3.1% for SSFP with 2.5 mg and 19.1% ± 6.0% for SSFP with 5 mg. The maximum SNR enhancement is earlier using 5 mg dose than 2.5 mg. There were greater than 15% increase in vessel lumen diameter throughout the 5 post-Isordil scans, with a greater than 20% increase in all but the first time point.

## Conclusion

Pre-scan Isordil administration improves coronary SNR by 20% for both GRE and SSFP imaging. 5 mg and 2.5 mg doses result in comparable vasodilation. For best SNR enhancement, imaging should be performed later post-Isordil if using 2.5 mg dose than 5 mg.
